# *microntology*: a lightweight, data-driven controlled vocabulary to describe earth’s microbial habitats

**DOI:** 10.1093/bioinformatics/btag343

**Published:** 2026-05-27

**Authors:** Anthony Fullam, P K Vishnu Prasoodanan, Michael Kuhn, Peer Bork, Thomas S B Schmidt

**Affiliations:** Molecular Systems Biology Unit, European Molecular Biology Laboratory, D-69117 Heidelberg, Germany; APC Microbiome and School of Medicine, University College Cork, Cork T12 Y337, Ireland; Molecular Systems Biology Unit, European Molecular Biology Laboratory, D-69117 Heidelberg, Germany; Molecular Systems Biology Unit, European Molecular Biology Laboratory, D-69117 Heidelberg, Germany; APC Microbiome and School of Medicine, University College Cork, Cork T12 Y337, Ireland

## Abstract

**Motivation:**

Data-enabled studies of microbial ecology and evolution depend on high-quality descriptions of microbial habitats, based on curated and consolidated vocabularies.

**Results:**

We introduce *microntology* v1.0, a pragmatic controlled vocabulary of 148 terms to describe microbial habitats and lifestyles, and provide manually curated *microntology* annotations for >300k metagenomic samples from public repositories.

**Availability:**

*microntology* controlled vocabulary terms and term hierarchies (doi: 10.5281/zenodo.19730167), and curated annotations for 305 626 metagenomic samples (doi: 10.5281/zenodo.18164252) are available via Zenodo and spire.embl.de/downloads. Underlying code is available via github.com/grp-schmidt/microntology and Zenodo (doi: 10.5281/zenodo.20323497). User feedback, suggestions and bug reports are welcome at github.com/grp-schmidt/microntology/issues.

## 1 Introduction

Data on microbial life continues to accrue in public repositories: at the time of writing, nearly 6 million amplicon sequencing and 1.2 million metagenomic experiments on microbial communities are freely accessible via the International Nucleotide Sequence Database Collaboration (INSDC, Karsch-Mizrachi *et al.* 2025), with rapid growth also in other ‘omic data types (Yurekten *et al.* 2024, Perez-Riverol *et al.* 2025). This wealth of information enables studies of microbial ecology and evolution at unprecedented *depth* (i.e. with deep readouts on individual samples or habitats) and *breadth* (across many samples, often across different habitats), facilitated by dedicated resources that integrate microbial data across Earth’s biomes, such as catalogues of genes (Coelho *et al.* 2022), gene families (Rodríguez Del Río *et al.* 2024), metabolic pathways (Kautsar *et al.* 2021), metagenome-assembled genomes (MAGs, Gurbich *et al.* 2023, Nayfach *et al.* 2021, Schmidt *et al.* 2024, Sun *et al.* 2026) or taxonomic profiles (Woodcroft *et al.* 2025, Matias Rodrigues *et al.* 2026).

A key challenge in navigating repositories and identifying datasets relevant to specific research questions is the varying availability of consistent contextual data, which is why INSDC databases require submissions to conform with MIxS standards by providing Minimum Information about Genome/Metagenome Sequences (MIGS/MIMS, Field *et al.* 2008), MAGs (MIMAGS, Bowers *et al.* 2017) or MARKer gene Sequences (MIMARKS, Yilmaz *et al.* 2011). These include descriptions of the broad-scale and local environmental context [formerly defined as annotation fields *environment (biome)* and *environment (feature)*], as well as the sampled environmental medium [*environment (material)*], with a recommendation to use terms from corresponding classes in the Environment Ontology (EnvO, Buttigieg *et al.* 2013, 2016). However, annotations are often incomplete, uninformative (using generic terms) or indeed erroneous (Kuhn *et al.* 2026) and require extensive curation and harmonization, provided via dedicated databases such as curatedMetagenomicData (Pasolli *et al.* 2017) or Metalog (Kuhn *et al.* 2026). Moreover, EnvO and other available controlled vocabularies to describe microbial habitats, such as Omnicrobe (Dérozier *et al.* 2023) or the GOLD Ecosystem Classification (Mukherjee *et al.* 2025) used for IMG/M resources (Chen *et al.* 2023), are designed towards maximum resolution, containing thousands of classes that can be difficult to navigate and summarise into broader categories of related terms. Here, we introduce *microntology*, a lightweight controlled vocabulary to describe microbial habitats and lifestyles that addresses these challenges and complements existing ontologies.

## 2 Results and discussion

*microntology* follows five core design principles: it is (i) data-driven, encompassing terms to describe frequent sample types in existing data; (ii) pragmatic and query-oriented, defining terms at intermediate resolutions to facilitate user access and browsing; (iii) shallow, with few hierarchical levels of classes; (iv) cross-linked between related terms from different hierarchical paths (e.g. a *lake* [MICRONT: 02010100] would also be considered a *lentic water body* [MICRONT: 02060100]); and (v) designed for parsimonious multi-tagging where individual samples are described by multiple independent terms, rather than a single highly detailed ‘best match’ term (see examples below).

**Figure 1:**
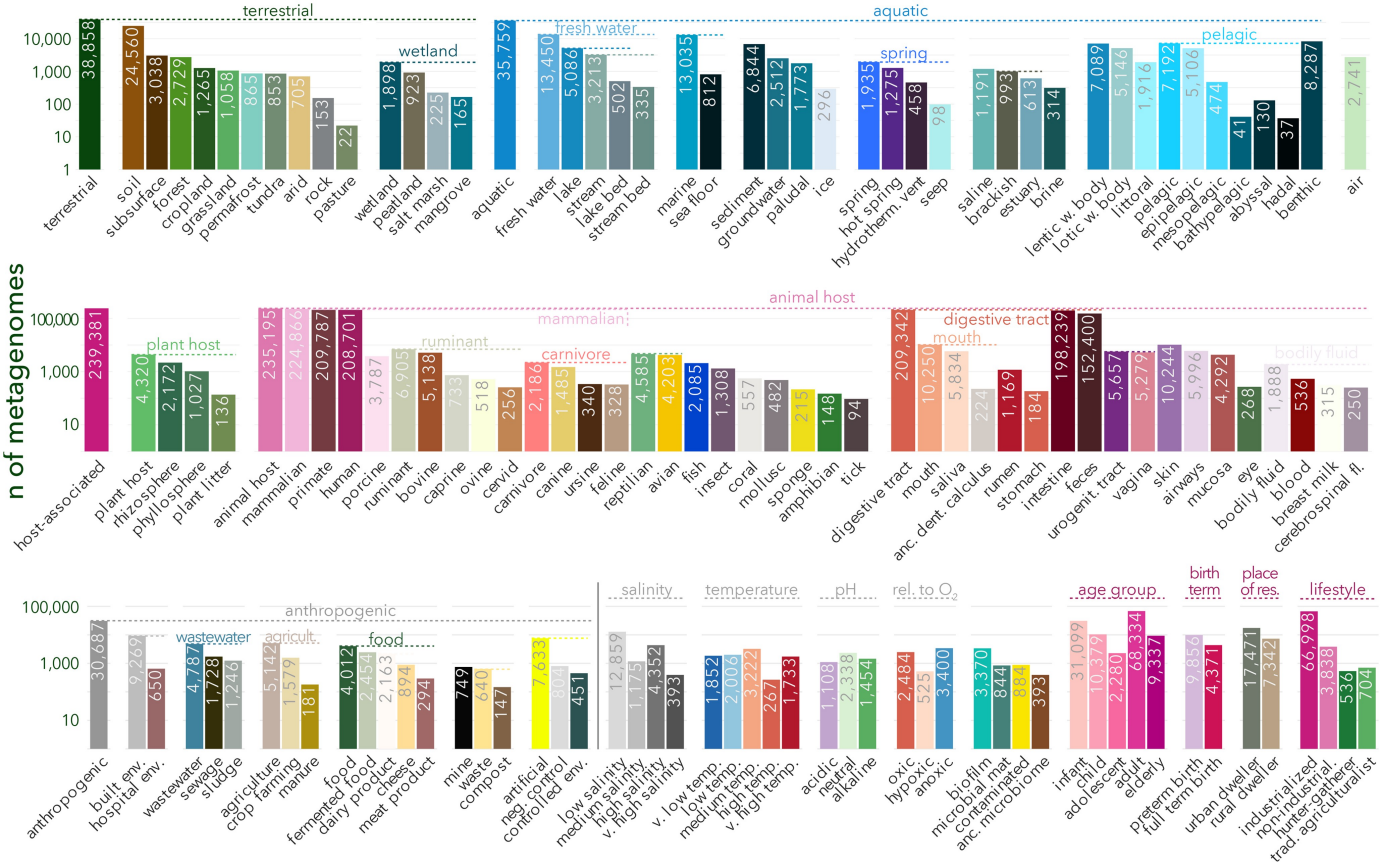
Overview of curated *microntology* annotations for 305 626 publicly available metagenomes. For each *microntology* term (categorical x axis), the number of annotated metagenomic samples (y axis) is shown. Categories are organized by sample group. Note that y axis scales differ between rows for improved visibility. As annotations follow hierarchical categories and individual samples are annotated with multiple tags (see main text), total counts do not sum to the total number of annotated metagenomes.

In v1.0, *microntology* encompasses a controlled vocabulary of 148 terms that fall into five broad habitat categories (terrestrial, aquatic, aerial, host-associated and anthropogenic environments) and additional categories that describe broad physicochemical properties (sample salinity, temperature, pH and oxygen level) which are often associated with microbial community structure in different habitats, morphology (biofilms), human host properties to further differentiate within this dominant sample group (age group, birth term, place of residence, non-industrialized lifestyles), animal host captivity status, and miscellaneous informative features such as (chemical) contamination or ancient (paleontological) sample provenance. Terms are moreover linked to matching classes in EnvO (Buttigieg *et al.* 2013, 2016), UBERON (Mungall *et al.* 2012) and other existing ontologies, as well as to NCBI Taxonomy IDs (Schoch *et al.* 2020) where applicable. An overview of *microntology* terms (and their frequency among currently available metagenomes, see below) is shown in Fig. 1; a full table including resolved hierarchies, cross-linked terms and definitions is available as Supplementary Table S1 and in Fullam *et al.* (2026a).

Major habitat categories are further resolved along parallel yet cross-linked paths. *Terrestrial* habitats (MICRONT: 01000000) can be described based on sample type (e.g. *soil, rock, subsurface*) and environmental context (*forest, grassland, tundra*, etc.). Following *microntology’s* underlying philosophy of using multiple descriptive tags, a forest soil sample would be annotated using the terms *soil* (MICRONT: 01010000) and *forest* (MICRONT: 01020400), rather than a specific term ‘forest soil’ as a subclass of one or the other. *Aquatic* habitats (MICRONT: 02000000) are further categorized based on general salinity ecosystem (*fresh water, brackish, marine, non-marine saline, brine*), water body type (*lentic* or *lotic*), broad water layer (*littoral, pelagic, benthic*), and dedicated categories describing *springs* (including *hydrothermal vents*), *ice*, and *groundwater* samples. Aquatic *sediments* are considered as a distinct subcategory, cross-linked to *benthic* features (*lake* and *stream beds*, *seafloor*). Descriptions of habitats at terrestrial-aquatic interfaces are resolved via both crosslinking of terms and multiple tagging: *groundwater* (MICRONT: 02070100) is considered an *aquatic* habitat, but automatically cross-tagged as *terrestrial* and *subsurface* (MICRONT: 01030000); *wetlands* (MICRONT: 01020700) are categorized as *terrestrial*, but cross-tagged as *lentic* (MICRONT: 02060100) and *paludal* (MICRONT: 02090000) water bodies; *soils* from intertidal zones or ocean coasts would in practice be co-tagged as *littoral* (MICRONT: 02080100) and *marine* (MICRONT: 02020000).

*Host-associated* samples (MICRONT: 03000000) are broadly split into *plant host* (MICRONT: 03010000) and *animal host* (MICRONT: 03020000) groups. The latter is further resolved based on broad host taxonomy (*mammalian, avian, sponge hosts*, etc), body site (*digestive tract, urogenital tract, skin, airways*, etc) and sample type (*bodily fluids* or *mucosa*). *Feces* (MICRONT: 03030141), *saliva* (MICRONT: 03030111) and *ancient dental calculus* (MICRONT: 03030112) are sample types that are treated as special cases, as they are unambiguously *intestinal* (MICRONT: 03030140) and *oral* samples (MICRONT: 03030110), respectively. Following *microntology’s* multi-tag approach, a dog fecal sample would be annotated as *canine* (MICRONT: 03020131) and *feces* (MICRONT : 03030141), whereas caecal contents of chicken would be considered as *avian* (MICRONT: 03020210) *intestine* (MICRONT: 03030140). For *human* (MICRONT: 03020111) samples, by far the most data-rich category in practice, additional information can be added by tagging age group (from *infant* to *elderly*), birth term (*full* or *preterm birth*), place of residence (*urban* or *rural*) and host lifestyle (e.g. *non-industrialized*).

The *anthropogenic* (MICRONT: 04000000) category includes engineered or directly human-impacted habitats, such as the *built environment* (MICRONT: 04020000), *agriculture*-associated environments (MICRONT: 04040000), *wastewater* (MICRONT: 04030000) and *food* (MICRONT: 04060000). *microntology* includes a special *artificial sample* category (MICRONT: 04010000) to flag mock communities or negative controls, but also to describe experimentally derived samples from raw environmental starting materials, e.g. using controlled environments, cell sorting or enrichment cultures. For example, microcosm experiments from lake sediments would be annotated as *lake bed* (MICRONT: 02010101), but qualified with a *controlled environment* (MICRONT: 04010300) tag.

We curated *microntology* annotations for 305 626 publicly available shotgun metagenomic samples from 1688 studies [Fig. 1; per-sample data available under (Fullam *et al.* 2026b)]. We initially included all metagenomes (*source: METAGENOMIC* and *strategy: WGS*) from projects containing ≥20 samples with ≥1M reads and ≥1Gpb sequencing depth, generated on Illumina machines or compatible platforms (DNBseq, BGIseq), that were available on the European Nucleotide Archive [ENA, O’Cathail *et al.* 2025) on 31st October 2024. From this initial list, datasets were excluded upon manual inspection if they were (i) misannotated amplicon or genomic sequencing data; (ii) artificial samples such as synthetic metagenomes or mock communities; (iii) obtained from *in vivo* models in a laboratory context, e.g. mouse experiments; (iv) enriched for viral particles or specific prokaryotic community members. Additional datasets were included on a per-study basis, in particular from IMG/M (Chen *et al.* 2023) and SPIRE (Schmidt *et al.* 2024). For 1501 studies (representing 95% of samples), ENA project IDs were matched to publications following a previously described approach (Schmidt *et al.* 2024, Kuhn *et al.* 2026). *microntology* terms were annotated in multiple steps (see Supplementary Discussion for details). First, for a subset of 129 904 samples, available manually curated information was parsed from Metalog (Kuhn *et al.* 2026) and matched to *microntology* tags. Next, we mapped tags to entries from ENA contextual data fields (such as environmental context, scientific name and host tax ID). Finally, for a subset of 163 studies and an additional 22 862 samples, *microntology* tags were manually added based on information in associated publications or additional (not automatically parsed) ENA contextual data fields.

In our survey (Fig. 1; see Supplementary Fig. S1 for an alternative view), publicly available metagenomic data remains strongly biased towards animal-associated habitats (235k samples, or 77% of total), and in particular to human (209k, 68.5%) and intestinal (198k, 65%) samples. However, the representation of other body sites (such as mouth, skin, airways and urogenital tract), non-human hosts (in particular bovine, porcine and avian), but also of soils (25k samples), fresh water (13.5k), ocean (13k), the built environment (9k) and food-associated habitats (4k) has increased significantly compared to recent surveys (e.g. Schmidt *et al.* 2024). We note that for additional descriptive categories, such as salinity, temperature, human age group or place of residence, annotation recall is necessarily limited, as the corresponding information is often not available in public repositories, nor can it be extracted from publications. In such instances, we annotated conservatively, i.e. assigning only tags unambiguously supported by publicly available data, without extrapolation.

*microntology* annotations have previously been used to facilitate data exploration e.g. in SPIRE v1 (Schmidt *et al.* 2024) and the Global Microbial SmORFs Catalogue (Duan *et al.* 2024), to benchmark global-scale microbial habitat unsupervised taxonomic clusterings and modeling (Kim *et al.* 2026), and to categorize the main reservoirs of discoverable microbial diversity in existing data (Prasoodanan Pk *et al.* 2026). The resource is continuously developed and iteratively refined, following the basic principles laid out above, i.e. driven by data availability and user requirements in practice, relying on multiple parsimonious tags to describe microbial habitats at an intermediate and pragmatic resolution. *microntology* is intended as a lightweight complement to established highly detailed ontologies such as EnvO (Buttigieg *et al.* 2016) or the GOLD Ecosystem Classification (Mukherjee *et al.* 2023) while retaining interoperable links, yet to provide broader coverage than project-specific vocabularies such as the Earth Microbiome Project (Thompson *et al.* 2017) or Microflora Danica ontologies (Singleton *et al.* 2026). By design, *microntology* describes microbial habitats at a resolution determined by the availability and scope of underlying data, in particular metagenomic samples. Therefore, *microntology* annotations can be browsed as a rough classification of datasets towards explorative analyses, e.g. to track microbial responses to rapidly changing conditions across Earth’s habitats. Other resources such as curatedMetagenomicData (Pasolli *et al.* 2017) or Metalog (Kuhn *et al.* 2026) provide detailed descriptions of individual samples and their ecological niches with detailed contextual data. In summary, *microntology* annotations may facilitate studies of microbial ecology and evolution at large scales, integrated across Earth’s microbial habitats and lifestyles.

## Supplementary Material

btag343_Supplementary_Data

## References

[btag343-B1] Bowers RM , Kyrpides NC, Stepanauskas R et al Minimum information about a single amplified genome (MISAG) and a metagenome-assembled genome (MIMAG) of bacteria and archaea. Nat Biotechnol 2017;35:725–31. 28787424 10.1038/nbt.3893 PMC6436528

[btag343-B2] Buttigieg P , Morrison N, Smith B, the ENVO Consortium et al The environment ontology: contextualising biological and biomedical entities. J Biomed Semant 2013;4:43. 10.1186/2041-1480-4-43 PMC3904460 24330602

[btag343-B3] Buttigieg PL , Pafilis E, Lewis SE et al The environment ontology in 2016: bridging domains with increased scope, semantic density, and interoperation. J Biomed Semant 2016;7:57. 10.1186/s13326-016-0097-6 PMC5035502 27664130

[btag343-B4] Chen I-MA , Chu K, Palaniappan K et al The IMG/M data management and analysis system v.7: content updates and new features. Nucleic Acids Res 2023;51:D723–D732. 36382399 10.1093/nar/gkac976 PMC9825475

[btag343-B5] Coelho LP , Alves R, Del Río ÁR et al Towards the biogeography of prokaryotic genes. Nature 2022;601:252–6. 34912116 10.1038/s41586-021-04233-4 PMC7613196

[btag343-B6] Dérozier S , Bossy R, Deléger L et al Omnicrobe, an open-access database of microbial habitats and phenotypes using a comprehensive text mining and data fusion approach. PLoS One 2023;18:e0272473. 36662691 10.1371/journal.pone.0272473 PMC9858090

[btag343-B7] Duan Y , Santos-Júnior CD, Schmidt TS et al A catalog of small proteins from the global microbiome. Nat Commun 2024;15:7563. 39214983 10.1038/s41467-024-51894-6 PMC11364881

[btag343-B8] Field D , Garrity G, Gray T et al The minimum information about a genome sequence (MIGS) specification. Nat Biotechnol 2008;26:541–7. 18464787 10.1038/nbt1360 PMC2409278

[btag343-B9] Fullam A , Prasoodanan PK V, Kuhn M et al microntology: a controlled vocabulary to describe Earth’s microbial habitats. 2026a. 10.5281/zenodo.18162128 PMC13259632 42203690

[btag343-B10] Fullam A , Prasoodanan PK V, Schmidt TSB et al microntology annotations of publicly available metagenomes in the European Nucleotide Archive. 2026b. 10.5281/zenodo.18164251

[btag343-B11] Gurbich TA , Almeida A, Beracochea M et al MGnify genomes: a resource for biome-specific microbial genome catalogues. J Mol Biol 2023;435:168016. 36806692 10.1016/j.jmb.2023.168016 PMC10318097

[btag343-B12] Karsch-Mizrachi I , Arita M, Burdett T et al The international nucleotide sequence database collaboration (INSDC): enhancing global participation. Nucleic Acids Res 2025;53:D62–D66. 39535044 10.1093/nar/gkae1058 PMC11701530

[btag343-B13] Kautsar SA , Blin K, Shaw S et al BiG-FAM: the biosynthetic gene cluster families database. Nucleic Acids Res 2021;49:D490–D497. 33010170 10.1093/nar/gkaa812 PMC7778980

[btag343-B14] Kim CY , Podlesny D, Schiller J et al Planetary microbiome structure and generalist-driven gene flow across disparate habitats. Cell 2026;189:2073–91.e21. 41666926 10.1016/j.cell.2025.12.051

[btag343-B15] Kuhn M , Schmidt TSB, Ferretti P et al Metalog: curated and harmonised contextual data for global metagenomics samples. Nucleic Acids Res 2026;54:D826–34. 10.1093/nar/gkaf1118 41171125 PMC12807751

[btag343-B16] Mukherjee S , Stamatis D, Li CT et al Genomes OnLine database (GOLD) v.10: new features and updates. Nucleic Acids Res 2025;53:D989–D997. 39498478 10.1093/nar/gkae1000 PMC11701667

[btag343-B24] Matias Rodrigues JF , Tackmann J, Malfertheiner L et al The MicrobeAtlas database: global trends and insights into Earth's microbial ecosystems. Cell 2026;189:2092–107.e17. 10.1016/j.cell.2026.01.021 41747730

[btag343-B17] Mukherjee S , Stamatis D, Li CT et al Twenty-five years of genomes OnLine database (GOLD): data updates and new features in v.9. Nucleic Acids Res 2023;51:D957–D963. 36318257 10.1093/nar/gkac974 PMC9825498

[btag343-B18] Mungall CJ , Torniai C, Gkoutos GV et al Uberon, an integrative multi-species anatomy ontology. Genome Biol 2012;13:R5. 22293552 10.1186/gb-2012-13-1-r5 PMC3334586

[btag343-B19] Nayfach S , Roux S, Seshadri R, IMG/M Data Consortium et al A genomic catalog of earth’s microbiomes. Nat Biotechnol 2021;39:499–509. 33169036 10.1038/s41587-020-0718-6 PMC8041624

[btag343-B20] O’Cathail C , Ahamed A, Burgin J et al The european nucleotide archive in 2024. Nucleic Acids Res 2025;53:D49–D55. 39558171 10.1093/nar/gkae975 PMC11701661

[btag343-B21] Pasolli E , Schiffer L, Manghi P et al Accessible, curated metagenomic data through ExperimentHub. Nat Methods 2017;14:1023–4. 29088129 10.1038/nmeth.4468 PMC5862039

[btag343-B22] Perez-Riverol Y , Bandla C, Kundu DJ et al The PRIDE database at 20 years: 2025 update. Nucleic Acids Res 2025;53:D543–D553. 39494541 10.1093/nar/gkae1011 PMC11701690

[btag343-B23] Prasoodanan Pk V , Maistrenko OM, Fullam A et al Unbinned contigs expand known diversity in the global microbiome. Nat Microbiol 2026;11:1463. 42045370 10.1038/s41564-026-02354-y PMC13171622

[btag343-B25] Rodríguez Del Río Á , Giner-Lamia J, Cantalapiedra CP et al Functional and evolutionary significance of unknown genes from uncultivated taxa. Nature 2024;626:377–84. 38109938 10.1038/s41586-023-06955-z PMC10849945

[btag343-B26] Schmidt TSB , Fullam A, Ferretti P et al SPIRE: a searchable, planetary-scale mIcrobiome REsource. Nucleic Acids Res 2024;52:D777–D783. 37897342 10.1093/nar/gkad943 PMC10767986

[btag343-B27] Schoch CL , Ciufo S, Domrachev M et al Ncbi taxonomy: a comprehensive update on curation, resources and tools. Database 2020;2020. 10.1093/database/baaa062 PMC7408187 32761142

[btag343-B28] Singleton CM , Jensen TBN, Delogu F et al The Microflora Danica atlas of Danish environmental microbiomes. Nature 2026;649:971–81. 10.1038/s41586-025-09794-2 41339548 PMC12823411

[btag343-B29] Sun Y , Chen Q, Fan G et al gcMeta 2025: a global repository of metagenome-assembled genomes enabling cross-ecosystem microbial discovery and function research. Nucleic Acids Res 2026;54:D724–D733. 41171134 10.1093/nar/gkaf1115 PMC12807734

[btag343-B30] Thompson LR , Sanders JG, McDonald D, The Earth Microbiome Project Consortium et al A communal catalogue reveals earth’s multiscale microbial diversity. Nature 2017;551:457–63. 29088705 10.1038/nature24621 PMC6192678

[btag343-B31] Woodcroft BJ , Aroney S, Zhao R et al Comprehensive taxonomic identification of microbial species in metagenomic data using SingleM and Sandpiper. Nat Biotechnol 2025. 10.1038/s41587-025-02738-1 40670710

[btag343-B32] Yilmaz P , Kottmann R, Field D et al Minimum information about a marker gene sequence (MIMARKS) and minimum information about any (x) sequence (MIxS) specifications. Nat Biotechnol 2011;29:415–20. 21552244 10.1038/nbt.1823 PMC3367316

[btag343-B33] Yurekten O , Payne T, Tejera N et al MetaboLights: open data repository for metabolomics. Nucleic Acids Res 2024;52:D640–D646. 37971328 10.1093/nar/gkad1045 PMC10767962

