# Supplementary material for: microntology: a lightweight, data-driven controlled vocabulary to describe earth’s microbial habitats
Source: Bioinformatics. 2026 May 27;42(6):btag343. doi: 10.1093/bioinformatics/btag343 (PMC13259632; doi:10.1093/bioinformatics/btag343)
Supplement: btag343_Supplementary_Data [file btag343_supplementary_data.zip › manuscript.microntology.supplement.r01.pdf]

Supplementary Discussion: curated annotation of publicly available samples with microntology terms

microntology annotations of publicly available metagenomic samples were obtained via a series of manual annotation and curation steps. The full workflow and underlying computer code are documented in the associated repository on GitHub (https://github.com/grp-schmidt/microntology).

Automated annotation based on manually curated information in Metalog.

The Metalog database (metalog.embl.de) contains manually curated and harmonized contextual data for more than 150,000 metagenomic samples (as of 04-2026). *microntology* annotations are obtained by mapping terms to data from specific Metalog fields, e.g. source material or environment, based on specific mapping tables. Additional annotations are derived from continuous data fields, e.g. age group is assigned based on curated information from Metalog fields “age (years)”, “age range (years)” and “age category”, or oxygen level categories based on “oxygen_uM” for environmental samples.

Automated annotation based on submitted ENA metadata.

For samples that are not (yet) curated in Metalog, sample-level metadata is directly parsed from the European Nucleotide Archive. *microntology* terms are mapped to free text in specific ENA data fields, such as “environment_material”, “isolation_source”, or to informative NCBI taxonomy in sample-level “scientific_name” descriptions. In particular, this includes steps to detect samples for exclusion from subsequent steps due to misannotations (e.g., microbial isolate sequencing samples or metagenome-assembled genomes, i.e. not shotgun metagenomes). In addition, ENA metadata fields containing relevant continuous variables (like oxygen concentration, salinity, pH, etc.) are lightly harmonized and then mapped onto respective ranges for corresponding *microntology* tags.

Manual annotation of individual samples and entire studies.

Where applicable, *microntology* terms are mapped to entire studies (by ENA project ID) and individual samples (by ENA biosample ID). This mapping is performed based on information extracted from underlying matched publications and/or contextual free text data downloaded from ENA directly.

Consolidation of term annotations.

In a final step, annotations from all above channels are consolidated additively, i.e. redundancies are removed, but individual samples can receive *microntology* annotation tags from various sources. Once a consolidated terms list is established, crosslinked terms for all encountered terms are added: e.g., MICRONT:02060100 (“lentic water body”) is added for each sample annotated with MICRONT:02010100 (“lake”). Finally, for each term, all parent terms are added non-redundantly, e.g. for MICRONT:03030141 (“feces”), terms MICRONT:03000000 (“host-associated”), MICRONT:03020000 (“animal host”), MICRONT:03030100 (“digestive tract”) and MICRONT:03030140 (“intestine”) are included. Each sample

is thus annotated with a full complement of relevant terms, making the resulting dataset fully searchable across different granularities.

Supplementary Figure

Figure S1. A habitat-resolved view of available metagenomic data. *microntology* annotations were further summarised to label each sample under a most descriptive category. In the treemap plot, each cell corresponds to an individual study (out of 1,688 total studies); cell size corresponds to the number of samples in a study in the focal habitat category.

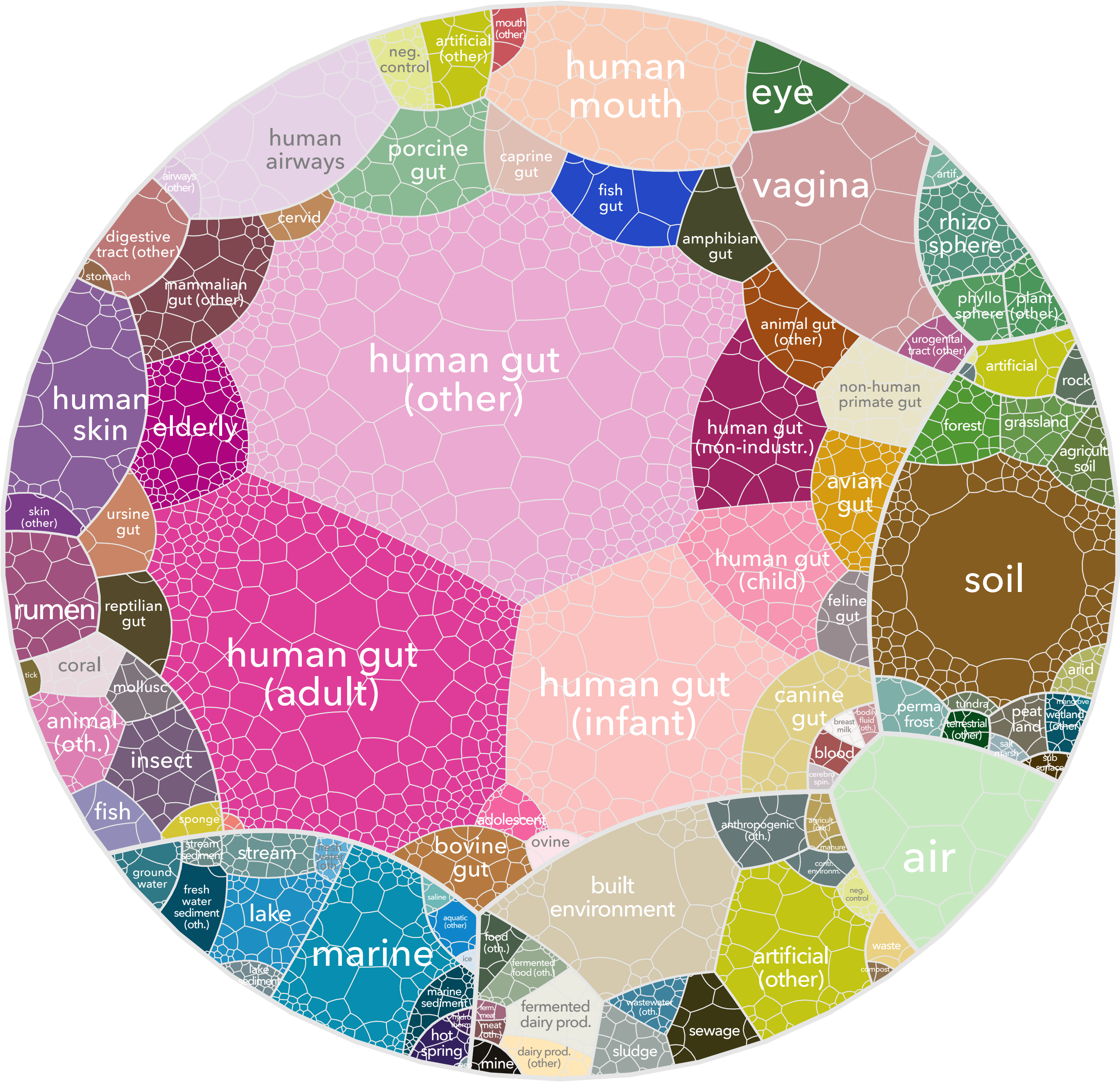

human mouth
mouth (other)
artificial (other)
neg. control
human airways
airways (other)
porcine gut
caprine gut
eye
vagina
fish gut
cervid
digestive tract (other)
stomach
mammalian gut (other)
amphibian gut
artif.
rhizo sphere
phyllo sphere
plant (other)
animal gut (other)
urogenital tract (other)
artificial
rock
human gut (other)
human skin
elderly
human gut (non-industr.)
non-human primate gut
forest
grassland
agricult soil
avian gut
skin (other)
ursine gut
soil
human gut (child)
feline gut
rumen
reptilian gut
human gut (adult)
human gut (infant)
coral
tick
mollusc
arid
canine gut
perma frost
tundra
terrestrial (other)
peat land
mangrove
wetland (other)
bodily fluid (oth.)
breast milk
blood
salt marsh
sub surface
animal (oth.)
insect
cerebro spin.
fish
sponge
adolescent
air
anthropogenic (oth.)
agricult. (oth.)
manure
ovine
bovine gut
built environment
stream sediment
stream
fresh water (oth.)
ground water
contr. environm.
neg. control
fresh water sediment (oth.)
lake
saline
aquatic (other)
marine
food (oth.)
ice
waste
compost
fermented food (oth.)
lake sediment
artificial (other)
marine sediment
fermented dairy prod.
wastewater (oth.)
ferm. meat
meat (oth.)
hot spring
sewage
sludge
dairy prod. (other)
mine
